# Cytosolic pressure provides a propulsive force comparable to actin polymerization during lamellipod protrusion

**DOI:** 10.1038/srep12314

**Published:** 2015-07-21

**Authors:** Daphne Manoussaki, William D. Shin, Clare M. Waterman, Richard S. Chadwick

**Affiliations:** 1 School of Electrical and Computer Eng., Technical University of Crete, Hania, Greece; 2 Cell Biology and Physiology Center, NHLBI/NIH, Bethesda, MD, USA; 3 Laboratory of Cellular Biology, Section on Auditory Mechanics, NIDCD/NIH

## Abstract

Does cytosolic pressure facilitate f-actin polymerization to push the leading edge of a cell forward during self-propelled motion? AFM force-distance (f-d) curves obtained from lamellipodia of live cells often exhibit a signal from which the tension, bending modulus, elastic modulus and thickness in the membrane-cortex complex can be estimated close to the contact point. These measurements permit an estimate of the cytosolic pressure via the canonical Laplace force balance. The deeper portion of the f-d curve allows estimation of the bulk modulus of the cytoskeleton after removal of the bottom effect artifact. These estimates of tension, pressure, cortex thickness and elastic moduli imply that cytosolic pressure both pushes the membrane forward and compresses the actin cortex rearward to facilitate f-actin polymerization. We also estimate that cytosolic pressure fluctuations, most likely induced by myosin, provide a propulsive force comparable to that provided by f-actin polymerization in a lamellipod.

The canonical view in cell mechanics is that an f-actin polymerization force pushes against the leading edge membrane of a lamellipod and causes protrusion^1^. The theoretical basis for the polymerization force is due to Hill & Kirschner^2^, who predicted that actin or microtubules could either push or pull against a load depending on whether the local monomer concentration is greater or less than a critical concentration, a quantity that is unknown and may not apply inside a cell because of capping and monomer sequestration. In any case, it is difficult to separate cytoplasmic pressure from polymerization as the origin of the pushing force since both types of pushing forces can coexist. While the actin polymerization force has been directly measured outside of a cell^3^, until recently there have been no such measurements inside a cell^4^. Farrell *et al.*^5^ recently reported an assay that involved pulling actin-filled tethers with an optical trap and interpreted the data using the Hill formalism. A conceptual difficulty with f-actin pushing the membrane is that it must detach from the cell membrane to enable a g-actin monomer to intercalate. A way around the difficulty has been to argue that statistical fluctuations in either the membrane position or the f-actin location will permit intercalation. These are the two forms of the Brownian ratchet mechanism^6^,^7^. However, there is a growing body of evidence that f-actin may not be in full contact with the leading edge and protrusion still occurs. For example, rapidly assembling f-actin dynamics occurs throughout a domain that is hundreds of nanometers wide as measured by qFSM^8^. Since polymerization can only occur at the end of an f-actin filament, filament ends should also be distributed over a domain that is hundreds of nanometers wide. If a filament end is not in contact with the leading edge, then it cannot exert a pushing force. Instead cytosolic pressure must do the pushing. Indeed a reduced number filament ends near the membrane was measured using cryo-EM^9^. Furthermore a rearward shift away from the membrane of a dorsal layer of f-actin was seen using dual-objective STORM^10^, which further reduces the number of f-actin ends in contact with the membrane. We also mention the absence of f-actin in contact with the membrane during bleb formation where it is clear that cytosolic pressure is dominant^11^. In what follows, we will analyse our data keeping in mind the model lamellipod having a ventral and dorsal cortical layer separated by a gap shown in Fig. 1, which is suggested by the images obtained by dual-objective STORM^10^.

## Results

### Indentation of the membrane/actin cortex by a paraboloid

The dorsal cell surface is assumed to be a flat horizontal surface indented by the AFM cantilever whose tip has the local shape of a paraboloid, as shown in Fig. 2. The paraboloid shape is used as the simplest model of an axisymmetric shape with finite radius of curvature 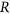. We assume that both tension and bending curvature elastically resist the probe tip. The contact mechanics is therefore governed by the membrane-plate equation


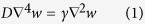


where *D* (N-m) is the bending modulus, *γ* (N/m) is the tension, and *w* is the vertical displacement induced by the probe.

It is easy to show that this contact problem can be reduced to the superposition of two previously solved problems: the indentation of an elastic half-space by a cone^12^ and the indentation of an elastic half-space by a flat-ended circular cylinder^13^. The mechanics problem is to determine the force *F* required to indent a membrane having tension *γ* (N/m) and bending modulus *D* (N-m) when the indentation distance is *δ*. The radial distribution of contact pressure *p*(*r*) and the contact radius *a* are auxiliary unknowns. The paraboloid 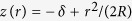 describes the shape of the tip in contact with the membrane as a function of the radial distance *r* when it is at distance *δ* lower than the upper cell surface before indentation. The same shape can be calculated as a result from a distribution of point forces acting over the contact area *πa*^2^, resulting from an unknown pressure distribution *p*(*ρ*). The resulting integral equation for the pressure-resulting indentation is


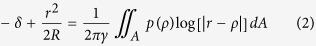


The log term is the axisymmetric point force solution of the plate-membrane equation, which governs the transverse displacement. We differentiate equation (2) with respect to *r* to obtain


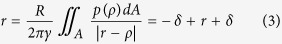


The right–hand side can be viewed as the superposition of the indentation by two shapes: a cone having a half-opening angle of *π*/4 (the −*δ* + *r* term) plus the pulling upward by a flat-ended cylinder (the *δ* term). The term 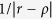 is the Green’s function for an elastic half-space. The pressure distribution is known for the cone and flat-ended cylinder problems in terms of the modulus of elasticity 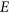 of the half-space. By looking at the integral equations for the half-space problems we can make the identification 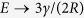 for an incompressible material. We then find the contact pressure acting on the membrane is (cf. ref. Johnson^14^).


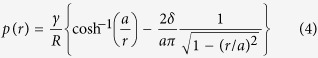


The net downward force acting on the membrane is


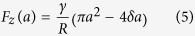


From classical contact mechanics^15^ the contact radius *a* can be found by setting 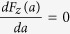, giving


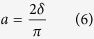


### Finding the cortical tension, bending modulus, elastic modulus and thickness from the AFM force-distance curve

The upward pushing force of the membrane tension acting on the tip must be balanced by the cantilever restoring force *kd*, where *k* is the spring constant of the cantilever and *d* is the deflection of the cantilever measured by the AFM laser signal. The vertical component of the tension force is 2*πrγ* cos*α* , where *α* is the angle between the tangent to the tip surface at the contact radius and the vertical direction. Thus


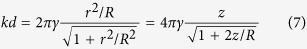


For the AFM piezo signal we need to relate *z* at the contact radius *a* to the distance below the horizontal line (original contact location) in Fig. 2,


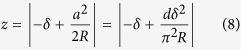


where we have used equation (6) to eliminate *a* in terms of *δ*. The force – indentation curve has an inflection at


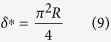


where the force is


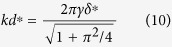


The ventral and dorsal cortices come together at this point to induce the curvature change. The membrane tension 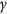 can be determined from equation (10) and the AFM measurements of (*k*, d^*^, δ^*^) (see Fig. 3). Note the probe radius *R* could not be measured directly, but was eliminated using equation (9).

The bending modulus can also be determined from δ^*^ and d^*^ as follows. Outside the contact region (*r* > *a*), we note that the axisymmetric solution Eq. 1 of is 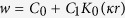 where 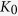 is the decaying modified Bessel function and 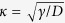. The two constants 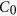 and 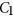 can be found by matching the known displacement and slope of the probe at 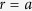. Since we require 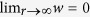, we must set 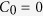, which yields the transcendental equation


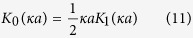


which has the root *κa* = 1.555265. This relation allows one to determine 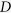 in terms of 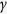 and 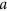. Once 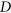 and 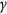 are determined we can estimate the cortex elastic modulus 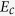 and thickness 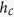 by combining Hooke’s law and elementary bending theory to obtain: 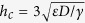 and 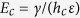, where the strain *ε* = 0.081 is calculated from the shape of the deformed surface.

### Comparison of AFM - determined membrane/cortex properties with other methods

PtK-1 rat-kangaroo kidney epithelial cells were plated on fibronectin coated glass bottom dishes and subjected to contact mode AFM indentation using gold coated silicon nitride four-sided pyramidal tip cantilevers using 5-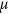
m ramps with up to 150 nm indentations at 1 Hz. This resulted in a stereotypical force-distance curve with a characteristic bump in the low force-distance regime when the cantilever tip was just coming in contact with the cell surface. Following the “bump” upon further distance indentation, a sharp rise in force was observed with increasing distance of indentation that is typical of cell indentation observed by others^16^,^17^. The values of tension shown for cells 1–5 in Table 1 are comparable to values obtained from keratocytes using the tether method^18^ and from fibroblasts using micropipette aspiration^11^. A kinematic method developed for bleb dynamics and based on plasma membrane viscosity yielded values 2 orders of magnitude smaller^19^. This may reflect the contribution of the cortex to membrane tension, which is periodically disrupted in blebbing cells. The substantially larger values of our bending modulus compared to others^5^ is most probably due to the fact that our experimental method is relatively gentle and determines the modulus of the combined membrane/cortex structure, instead of the membrane alone. It may be that the other methods that pull tethers using an optical trap or aspirate a cell into a micropipette disrupt the membrane cortex bonds.

### Determination of cell height and cytoskeletal bulk elasticity

With the ability to measure membrane tension at the leading edge, we then sought to determine if distinct actin structures within the leading edge exhibited distinct mechanical properties, and how the mechanical properties contribute to the overall balance of forces. To visualize we transfected PTK1 cells with GFP-tagged F-tractin (provided by Mike Schell, Uniformed Services University of the Heath Sciences, Bethesda, MD, USA) as a marker of actin cytoskeletal filaments and mCherry paxillin as a marker of integrin-based focal adhesions. Simultaneous live-cell fluorescence microscopy and AFM allowed us to choose specific cytoskeletal structures in the leading edge for AFM analysis. In order to analyze our data, we derived additional parameters to allow us to test this hypothesis. The parameters required for the balance of forces at the leading edge are membrane tension, cortical elastic modulus, cortex thickness, bulk cytoskeletal elastic modulus, cell height, cytosolic pressure, and the distance between the leading edge and a focal adhesion. Cell height and bulk modulus are obtained by fitting the deeper region of the force-distance curve (red portion of Fig. 3) to the BECC model of contact mechanics^16^. Cytosolic pressure, membrane thermal fluctuation mean amplitude, dendritic actin network bending thermal fluctuation mean amplitude, and dorsal and ventral actin polymerization zone width are estimated in the following subsections. We also calculate the probability that a peak fluctuation amplitude will exceed the size of a G-actin monomer.

### Estimation of cytosolic and polymerization pressures

**Figure 1:**
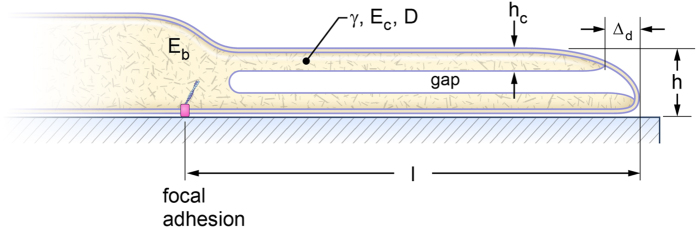
Model lamellipod suggested by dual objective STORM images^10^. Parameters are defined in text.

**Figure 2:**
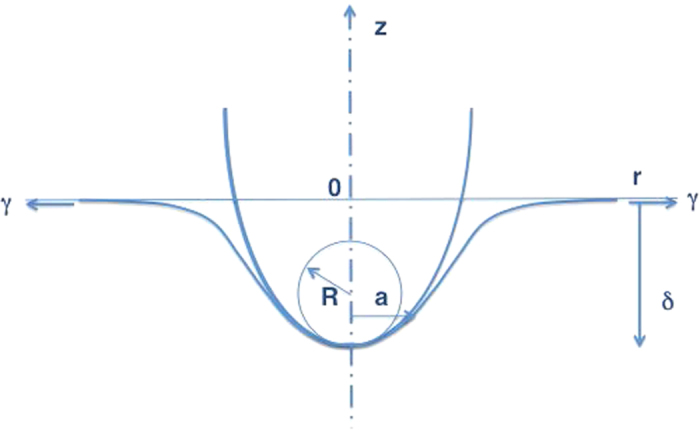
Geometry of membrane/actin cortex indentation by a paraboloid. Note space under dorsal surface is the gap shown in Fig. 1.

**Figure 3:**
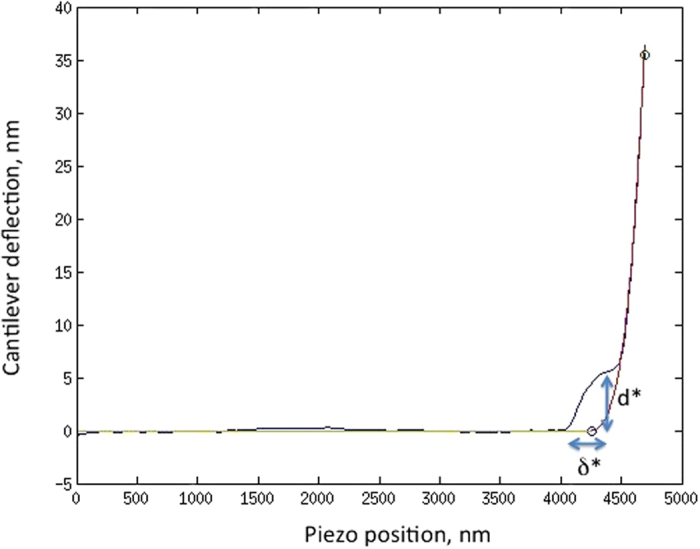
AFM deflection-indentation curve of a live PtK-1 cell illustrating membrane tension parameters. Cantilever deflection (nm) shown on *y*-axis. Piezo position (nm) shown on *x*-axis. Tension is determined from equation (10) using values of d^*^ and δ^*^ measured after estimating the locations of the contact point and the inflection point. Red line shows fit using Sneddon cone indentation theory^12^ with bottom artifact correction^16^ to determine the bulk modulus *E*_*b*_.

Considering the model in Fig. 1, the protrusion forces acting at the edge to the right are the cytosolic pressure *p* and the effective pressure due to actin polymerization *p*_*f*_. The resisting force acting to the left is due the membrane cortex tension *γ*. The force balance requires 
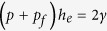
, where *h*_*e*_ is thickness of the lamellipod at its edge. We estimate *p*_*f*_ from the optical trap assay of Cojoc *et al.*^4^ who measured a net propulsive force of 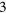
pN in filopodia with a diameter of 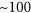 nm. The polymerization force is the sum of the net propulsive 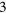 pN and the 15 pN membrane resistance force measured in a retracting filopod by Bornschloegl *et al.*^20^. We can achieve independent estimates of the polymerization pressure and the cytosolic pressure by using a filopod model based on EM images of Medalia *et al.*^21^, where it is clear that all the actin is polymerized in its tightly packed bundled core, i.e. there is no cortex layer and no space for fluid to generate a cytosolic pressure compared to the relatively sparce distribution of polymerized actin in a lamellipod. Therefore the effective polymerization pressure in the filopod measurement of Cojoc *et al.* is (15 + 3)pN/(*π*/4 ⋅ 100 nm^2^) ≈ 2.29 kPa.

However, in the lamellipod model (Fig. 1) only the ventral cortex is in contact with the membrane. The effective polymerization pressure in a lamellipod is therefore *p*_*f*_  ∼  2.29 *h*_*c*_/*h*_*e*_ kPa. To estimate *h*_*e*_ ∼ 300 nm we solved Eq. 1 with pressure loading with clamped conditions at the lamella end and free conditions at the edge. We assumed an unpressurized tapered shape seen by cryo EM^9^ with the height reduced from 200 nm to 100 nm at the edge over a distance of 1 *μ*m. Values of cytosolic and polymerization pressure are computed for each cell in Table 1.

### Estimation of membrane mean fluctuation amplitude

**Figure 4:**
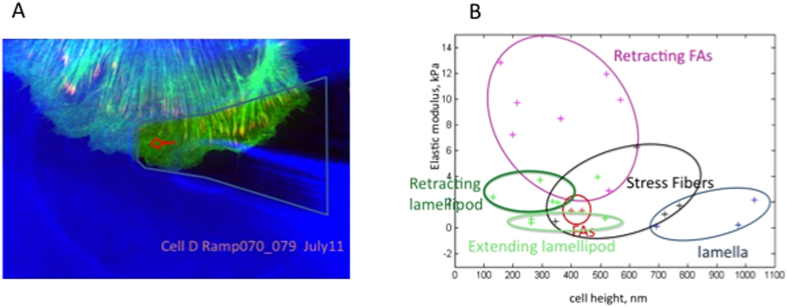
A: Image of PtK-1 lamellipod expressing GFP F-tractin as a marker of actin filaments and mApple paxillin as a marker of focal adhesions. Postion of cantilever tip can be located in each cell image. **B**: Bulk elastic modulus (kPa) vs cell height (nm) for various cell regions. Cell height determinded as part of fit to Sneddon model^12^ with bottom effect artifact correction that was previously validated^16^. Data collected from 5 Ptk-1 cells. FA-focal adhesion. Cytoskeletal elasticity depends specific locations within the lamellipod and whether it is extending or retracting.

Here we retrace the calculations of Mogilner & Oster^6^ but incorporate the AFM measurement of membrane tension reported in this study (Table 1) and cytoskeletal elastic modulus and cell height (Fig. 4B). These authors use the membrane fluctuation theory of Sackmann^22^ that estimates the membrane fluctuation amplitude from the formula


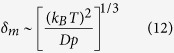


where *k*_*b*_*T* is the Boltzmann constant times temperature = 4.1 pN-nm, *D* is the membrane bending rigidity and *p* is the cytosolic pressure. Values of *δ*_*m*_ are given for each cell in Table 1 based on a factor of 10 smaller *D*, which is intended to mimic a membrane stripped of its cortex.

### Estimation of leading edge dendritic actin network mean bending fluctuation amplitude

We can use the theory for thermal tuning of cantilevers, based on the equipartition of energy theorem to estimate the flexural vibration amplitude *a*_*f*_ of the leading edge of f-actin having length *l* beyond the last branch point of an actin filament having diameter *b*.


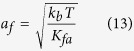


where *K*_*fa*_ is the stiffness constant of the dendritic f—actin network. From the deflection formula for a uniformly loaded cantilevered beam


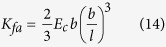


where *E*_*c*_ is the cortical elastic modulus of the leading edge. The the gap created by this amplitude is 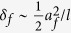.

Taking *b* = 15 nm from super resultion light microscopy^10^, and *l* = 100 nm from cryo EM^9^, we calculate the single filament gap *δ*_*f*_ due to flexural vibrations of a single filament shown in Table 1. The persistence length *L*_*p*_ has also been computed for each cell from the relation 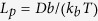 and listed in Table 1. The persistence length metric is often used in the analysis of *in vitro* images of filaments^23^.

### Estimation of polymerization zone width generated by cytosolic pressure

Consider the ventral cortex modeled as a poroelastic gel having length 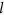 fixed at one end by focal adhesions and initially in contact with the membrane at the leading edge. We assume that cytosolic pressure *p* acts on all faces of the gel region except at the focal adhesion end which fixes that end, and we also assume that any membrane attachments are weak so that separation can occur at the leading edge. On the timescale of the edge protrusion or retraction cycle (∼10 sec) pressure does not have time to equalize inside the gel with the pressure on its boundaries due the hindered percolation of the cytosol through the gel. Indeed, such permeability-limited percolation leading to pressure non-equilibration has been previously noted as being likely to contribute to leading edge protrusion^24^. From elasticity theory, the compression of the cytoskeletal gel network having an elastic modulus *E*_*c*_, will create a gap between the membrane and actin allowing polymerization to take place in a zone having a width Δ_*v*_ given by


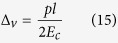


The calculated values for each cell are given in Table 1. A similar formula can be obtained for the dorsal actin cortex which we assume to be in series with the bulk cytoskeleton and have elasticity *E*_*b*_. The larger dorsal gap Δ_*d*_ results from the softer bulk cytoskeletal spring having an *E*_*b*_ = 5 kPa.

### The probability of membrane amplitude fluctuation peaks exceeding the monomer size

The amplitude probability density distribution function is classically known for the Brownian motion of a damped oscillator^25^. At long times, the probability density function is a Gaussian, so the probability *P* that a fluctuation amplitude is greater than or equal to *x* if the equipartition value is *a* is given by


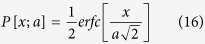


Erfc denotes the complimentary error function. The formula predicts that a g-actin monomer larger than the mean gap predicted by thermal equilibrium can still intercalate with a finite probability. The maximal dimension of an ellipsoidal model of a g-actin monomer can be estimated form its crystal structure to be 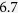 nm, while the same dimension is estimated as 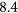 nm in solution using dynamic light scattering^26^. Taking *x* = 7 nm, recognizing that the solution monomer maximal dimension may be slightly larger than its crystallized value, but substantially larger than the usual value of 2.7 nm used in the Brownian ratchet literature^6^ based on the assembled helix repeat, and *a* = 7.3 nm for the mean value of membrane fluctuation amplitude given by the mean value of *δ*_*m*_ in Table 1 gives *P* = 0.169.

Other probabilities based on the means of *δ*_*f*_, Δ_*v*_, and Δ_*d*_ are 0.288, 0.381, and 0.481 respectively. We take notice that 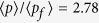 is close to the ratio of probability of intercalation into the mean ventral gap caused by cytosolic pressure, to the probabilty of intercalation into the mean gap caused by membrane fluctuations.

## Discussion

Novel analysis of AFM force-indentation curves presented here has allowed us to measure membrane/cortex tension, cortical membrane bending rigidity, cortical elastic modulus and cortical thickness at the leading edge of a lamellipod without the need for pulling tethers using an optical trap set-up or by micropipette suction. Both these previous methods are much harsher perturbations to the cell. The determination of tension at the same spatial location as the measurement of cytoskeletal bulk elasticity was achieved using a hybrid spinning disk/AFM set-up. Our microscope permitted us to examine the relationship between measured tension, calculated cytosolic pressure, and elastic moduli via a force balance. Although our measurements were made by probing the cell in a direction perpendicular to its protrusion direction we believe this is still meaningful to determine the local isotropic cytosolic pressure at the leading edge that provides the protrusive force. We could also estimate the effective actin polymerization pressure at the leading edge of a lamellipod based on the model shown in Fig. 1 and previous optical trap measurements by others^4^ on filipodia. We found that these two sources of propulsive force are comparable in a lamellipod. Our results also suggest that cytosolic pressure fluctuations facilitate actin polymerization by increasing the width of the polymerization zone. Our estimated zone of polymerization is consistent with previous actin dynamics imaging, as well as tomographic cryo – EM images showing a decreasing number of f-actin ends as the membrane is approached. Thus we conclude that pressure fluctuations provide a propulsive force comparable to actin polymerization, while also increasing the probability that g-actin intercalation can occur.

## Materials and Methods

### Imaging

The base plate of an AFM (Bioscope II, Bruker Instruments) was placed on an inverted microscope (Ti-E, Nikon). 488 nm and 561 nm excitation wavelength from a laser source (MLC 400, Agilent Technologies) was directed via fiber optics to a spinning disk confocal (CSU-X1, Yokogawa). Images were collected using a Plan APO VC 100 × 1.40 NA Phase objective on a high speed camera (Neo sCMOS, Andor Technology). Camera and peripherals were controlled through Metamorph software (Downingtown, PA). To minimize vibration induced from the disk rotation of the Yokogawa scan head, the CSU-X1 was mounted and aligned such that the scan head and microscope were not in direct contact. The placement of the AFM on the microscope was such that the AFM cantilever was in the microscope light path.

### Cells

PtK-1 cells were plated in a DMEM/Ham’s F-12 50/50 mix, supplemented with 10% fetal bovine serum on FN coated 1.5 glass-bottom dishes (WillCo-dish). Cells were transfected (Amaxa nucleofector), following the manufacturer’s protocol.

### Atomic Force Microscopy

Sharp tip contact mode AFM was used with BECC bottom correction^16^ for Young’s modulus and cell height determination. A new AFM model for membrane/cortex mechanical properties determination is presented here for indentation by a sharp tip with finite radius of curvature. Gold coated silicon nitride pyramidal tip MLTC cantilevers (Bruker Nano) were used with a spring constant 0.0365 N/m, determined by thermal fluctuation method. Ten ramps were averaged at a given location on the cell. The ramp rate was 1/sec.

## Additional Information

**How to cite this article**: Manoussaki, D. *et al.* Cytosolic pressure provides a propulsive force comparable to actin polymerization during lamellipod protrusion. *Sci. Rep.* **5**, 12314; doi: 10.1038/srep12314 (2015).

## Figures and Tables

**Table 1:** Data collected from 5 live PtK-1 cells using a cantilever with stiffness *k*=0.0365 N/m.

	Cell 1	Cell 2	Cell 3	Cell 4	Cell 5	Ref. 10	Ref. 11	Ref. 17	Ref. 18
d* (nm)	2.4	6.0	6.0	4.5	5.0				
δ^*^ (nm)	116	185	312	188	143				
*R* (nm)	47	75	126	76	58				
*γ* (pN/*μ*m)	224	350	208	259	378		414	280	6
*D* (*k*_*b*_*T*)	124	491	830	375	316				
*L*_*p*_ (*μm*)	1.9	7.4	12.5	5.6	4.7				15.0^23^
*h*_*c*_ (nm)	40.5	64.5	109	65.6	49.9	30–40			
*E*_*c*_ (kPa)	68.7	67.4	23.7	49.0	94.1				
*P*_*f*_ (kPa)	0.31	0.49	0.83	0.50	0.38				
*P* (kPa)	1.18	1.84	0.56	1.23	2.14				
Δ_*v*_ (nm)	17.2	27.3	23.6	25.1	22.7				
Δ_*d*_ (nm)	126.8	197.7	67.7	135.5	225.0				
*δ*_*m*_ (nm)	10.5	5.7	7.1	7.1	6.3				
*δ*_*f*_ (nm)	8.8	9.0	25.6	12.4	6.5				

Membrane tension determined using equation (10). Bending modulus can be calculated from the *κ* root (see text above), the membrane tension and equation (6). Calculation of all of other entries are described in text.
